# Biochemical markers in vascular cognitive impairment associated with subcortical small vessel disease - A consensus report

**DOI:** 10.1186/s12883-017-0877-3

**Published:** 2017-05-23

**Authors:** A. Wallin, E. Kapaki, M. Boban, S. Engelborghs, D. M. Hermann, B. Huisa, M. Jonsson, M. G. Kramberger, L. Lossi, B. Malojcic, S. Mehrabian, A. Merighi, E. B. Mukaetova-Ladinska, G. P. Paraskevas, B. O. Popescu, R. Ravid, L. Traykov, G. Tsivgoulis, G. Weinstein, A. Korczyn, M. Bjerke, G. Rosenberg

**Affiliations:** 1 Department of Psychiatry and Neurochemistry, Institute of Neuroscience and Physiology, Sahlgrenska Academy at the University of Gothenburg, Mölndal, Sweden; 2 1st Department of Neurology, Eginition Hospital, Medical School, National and Kapodistrian University of Athens, Athens, Greece; 3 Department of Neurology, University Hospital Centre Zagreb, Medical School, University of Zagreb, Zagreb, Croatia; 4 Memory Clinic and Department of Neurology, Hospital Network Antwerp (ZNA) Middelheim and HogeBeuken, Antwerp, Belgium; 5 Reference Center for Biological Markers of Dementia, Department of Biomedical Sciences, Institute Born-Bunge, University of Antwerp, Antwerp, Belgium; 6 Department of Neurology, University Hospital Essen, Essen, Germany; 7 Department of Neurology, University of California, Irvine, California USA; 8 Department of Neurology, University Medical Center Ljubljana, Ljubljana, Slovenia; 9 Department of Veterinary Sciences, University of Turin, Turin, Italy; 10 Department of Neurology, University Hospital “Alexandrovska”, Medical University, Sofia, Bulgaria; 11 Institute of Neuroscience, Campus for Ageing and Vitality, Newcastle University, Newcastle upon Tyne, NE4 5PL UK; 12 Department of Neurology, Colentina Clinical Hospital, School of Medicine, ‘Carol Davila’ University of Medicine and Pharmacy, Bucharest, Romania; 13 Brain Bank Consultants, Amsterdam, The Netherlands; 14 2nd Department of Neurology, Attikon Hospital, Medical School, National and Kapodistrian University of Athens, Athens, Greece; 15 School of Public Health, University of Haifa, Haifa, Israel; 16 Department of Neurology, Sackler School of Medicine, Tel Aviv University, Tel Aviv, Israel; 17 University of New Mexico Health Sciences Center, Albuquerque, NM 87131 USA; 18 Memory Clinic at Department of Neuropsychiatry, Sahlgrenska University Hospital, Institute of Neuroscience and Physiology at Sahlgrenska Academy, University of Gothenburg, Wallinsgatan 6, SE-431 41 Mölndal, Sweden

**Keywords:** Vascular cognitive impairment, Subcortical small vessel disease, Biomarkers, Blood, CSF, Alzheimer’s disease, Mixed type dementia, Dementia

## Abstract

**Background:**

Vascular cognitive impairment (VCI) is a heterogeneous entity with multiple aetiologies, all linked to underlying vascular disease. Among these, VCI related to subcortical small vessel disease (SSVD) is emerging as a major homogeneous subtype. Its progressive course raises the need for biomarker identification and/or development for adequate therapeutic interventions to be tested. In order to shed light in the current status on biochemical markers for VCI-SSVD, experts in field reviewed the recent evidence and literature data.

**Method:**

The group conducted a comprehensive search on Medline, PubMed and Embase databases for studies published until 15.01.2017. The proposal on current status of biochemical markers in VCI-SSVD was reviewed by all co-authors and the draft was repeatedly circulated and discussed before it was finalized.

**Results:**

This review identifies a large number of biochemical markers derived from CSF and blood. There is a considerable overlap of VCI-SSVD clinical symptoms with those of Alzheimer’s disease (AD). Although most of the published studies are small and their findings remain to be replicated in larger cohorts, several biomarkers have shown promise in separating VCI-SSVD from AD. These promising biomarkers are closely linked to underlying SSVD pathophysiology, namely disruption of blood-CSF and blood–brain barriers (BCB-BBB) and breakdown of white matter myelinated fibres and extracellular matrix, as well as blood and brain inflammation. The leading biomarker candidates are: elevated CSF/blood albumin ratio, which reflects BCB/BBB disruption; altered CSF matrix metalloproteinases, reflecting extracellular matrix breakdown; CSF neurofilment as a marker of axonal damage, and possibly blood inflammatory cytokines and adhesion molecules. The suggested SSVD biomarker deviations contrasts the characteristic CSF profile in AD, i.e. depletion of amyloid beta peptide and increased phosphorylated and total tau.

**Conclusions:**

Combining SSVD and AD biomarkers may provide a powerful tool to identify with greater precision appropriate patients for clinical trials of more homogeneous dementia populations. Thereby, biomarkers might promote therapeutic progress not only in VCI-SSVD, but also in AD.

## Background

There are not yet efficient ways of treating or preventing dementia disorders of which Alzheimer’s disease (AD) remains the most common target for therapeutic interventions. In contrast, only few clinical pharmacological trials have been conducted in subjects with vascular cognitive impairment (VCI). The lack of treatment success in VCI may be largely due to the heterogeneity of cerebrovascular diseases, with the majority of VCI clinical trials being performed in stroke patients. Only few clinical trials have been performed in patients with subcortical small vessel disease (SSVD), a common, fairly homogeneous, but often under-recognized type of VCI (VCI-SSVD) [1–4]. The clinical phenotype of combined AD and vascular pathologies is often referred to as mixed type dementia (MD). The overlap of SSVD, AD and normal aging makes the underlying clinical diagnosis challenging, and it may lead to misclassification of patients in treatment trials. One way of moving the field forward is to be more specific about disease definitions along the AD – SSVD axis. This, in turn, will allow for refinements of diagnostic criteria with more sharply defined patient cohorts.

## Method

A focused meeting on VCI-SSVD biochemical markers was held as a part of the 9th International Congress on Vascular Dementia in Ljubljana, Slovenia, on 18 October, 2015. Experts in this field reviewed the current evidence and literature data. For the purpose of this narrative review, we conducted a comprehensive search on Medline, PubMed and Embase databases for studies published until 15.01.2017. The key words used in the current search were: subcortical small vessel disease, vascular dementia, vascular cognitive impairment, Alzheimer’s disease, Binswanger’s disease, biomarker(s), cerebrospinal fluid, blood, serum, plasma, blood brain barrier, white matter, genetics, tau protein(s), amyloid, inflammation. We critically reviewed all abstracts and obtained the full text of relevant papers. We grouped the identified relevant subcortical small vessel disease biomarkers into themes and report the major relevant findings. The proposal on current status of biochemical markers in VCI-SSVD was reviewed by the group, including additional experts who did not attend the meeting. The draft was repeatedly circulated and discussed before it was finalized.

### Types of cerebral small vessel diseases, other vascular lesions and Alzheimer pathology

Cerebral SSVD refers to pathological processes affecting a spectrum of subcortical vascular changes visible on Computed Tomography/Magnetic Resonance Imaging (CT/MRI) as white matter lesions (WML), lacunes and cerebral microbleeds. Underlying vascular pathologies are arteriolosclerosis, lipohyalinosis, fibroid necrosis, oedema and damage to the blood-cerebrospinal fluid and blood–brain barriers (BCB/BBB), the latter resulting in chronic leakage of fluid and macromolecules in the white matter and inflammation (reviewed by Kalaria, 2016) [5]. Although SSVD pathogenesis remains poorly understood, it is an age-related condition, associated with a number of risk factors including systemic hypertension [6–9], chronic kidney disease [10, 11], smoking [12], metabolic syndrome [8, 13, 14], osteoporosis [15], chronic obstructive pulmonary disease [16] and sleep-apnoea syndrome [17]. The SSVD clinical manifestations are largely due to complete (lacunes) or incomplete (WML) infarction(s) and microbleeds resulting in cognitive, motor and mood disturbances and eventually functional disability [2], although same lesions might appear in cognitively intact persons during normal ageing. In patients with hypertension and WML, cerebral blood flow autoregulation is restricted, resulting in lack of physiological vasodilation during times of increased oxygen and nutrient needs. This renders the brain vulnerable to ischemic hypoperfusion, particularly in the watershed regions of the white matter [18].

Clinico-pathological studies have associated the characteristic clinical symptoms of VCI-SSVD (i.e. presence of motor and executive slowing, forgetfulness and dysarthria) with the bidirectional disruption of pathways connecting the prefrontal cortex with the basal ganglia and thalamus [2]. As opposed to other vascular subtypes, VCI-SSVD patients presents with a slow progressive course, which may mimic AD. On the other hand, late-onset AD patients frequently have significant SSVD burden, not necessarily related to their amyloid load [19]. The disruption of white matter network may be mediated via the effect of the small vessel pathology, rather than amyloid deposits [20]. A recent study showed age at onset as a crucial factor that determines distinct features in subcortical VCI patients, such as pathologic burden, structural changes and cognitive function. Early onset subcortical vascular cognitive impairment was reported to be associated with more lacunes, more severe frontal structural network disruption and more affected frontal executive functions. In contrast, later onset VCI-SSVD shows more pronounced amyloid burden, cortical and hippocampal atrophy [21]. The genetic component of VCI-SSVD is supported by the presence of monogenic forms representing a small portion of VCI-SSVD cases [22].

### Biochemical markers

Although several studies using biochemical markers have been completed and reviewed in AD, such information is still lacking for VCI-SSVD. Here we review studies using a broad range of biochemical markers from cerebrospinal fluid (CSF), plasma or serum in VCI-SSVD. Our primary question is whether they are useful to identify VCI-SSVD. We also review whether there are overlapping and differentiating findings between VCI-SSVD and AD.

### Blood-CSF/blood–brain barriers (BCB/BBB)

The BCB/BBB are highly selective permeability barriers that separate the circulating blood from the brain. The function and structure of BCB/BBB alter with aging. Disruption of BCB/BBB function, followed by blood-to-brain extravasation of circulating neuroinflammatory molecules, may increase the risk of brain injury. This may be an important factor for disease progression in both VCI and AD [23], and increasing evidence, also from pre-clinical translational studies, indicates that dysfunction of the BCB/BBB may play a significant role in the pathogenesis of vascular dementia [24–26]. The CSF/serum albumin quotient (QA) is the gold-standard measure of BCB/BBB integrity, with increases in this ratio indicating increased permeability. QA requires the measurement of albumin in CSF and serum collected concurrently [27, 28]. Advancing age is associated with increased BCB/BBB permeability, which is further increased in patients with VCI (as compared to AD) and with worsening of WML [29]. Altered BCB/BBB has consistently been reported in VCI-SSVD patients, and it is thought to contribute to the pathogenic process in AD [30–34].

Several lines of evidence suggested that the astrocytic protein S100β is a potentially useful peripheral marker of BCB/BBB permeability [35]. S100β can be released from injured astrocytes and enter the extracellular space and hence the bloodstream. This protein is elevated in radiologically defined SSVD [36, 37]. A positive correlation of S100β levels with the severity of depression, a common symptom in SSVD, has also been reported [38]. Changes in BCB/BBB function correlate strongly with an increase of two other proteins, namely glial fibrillary acidic protein (GFAP) and neuron specific enolase (NSE) in serum, indicating BCB/BBB leakage [39]. However, they are both unspecific biological markers in dementia disorders [40–42].

### Inflammatory and glial activation markers

Both the innate immune system and systematic inflammation have central roles in the pathophysiology of cognitive impairment [43, 44]. While many molecules within the inflammatory pathway are likely to be involved, only few of them have been investigated in the context of VCI-SSVD.

*C reactive protein* (CRP), a biomarker of systemic inflammation, is perhaps the most extensively studied circulatory biomarker of cerebral SSVD pathology. Although its association with SSVD (particularly in the presence of WML) is inconclusive [45–48], several studies have suggested that elevated peripheral CRP level increases the risk of VCI, but not AD [49, 50]. In addition, elevated serum CRP appears to be consistently associated with measures of white matter integrity [51, 52], whereas a rapid decline in CRP levels predicts a healthier white matter microstructure [53].

*Interleukins* (ILs) are a group of cytokines participating in the regulation of the immune response. A classification that proves useful in clinical practice (outside of structural biology) divides immunological cytokines into type 1 (IFN-γ, TNFα, TNFβ, IL-2, and IL-12-b), that enhances cellular immune responses and type 2 (TGF-β, IL-4, IL-5, IL-6, IL-10 and IL-13), which controls antibody responses. In the vasculature, interleukin-6 (IL-6) is secreted as a pro-inflammatory cytokine by *tunica muscularis* cells of the blood vessels. As a response to IL-6, the liver synthesizes the CRP. Although the majority of studies reported a positive association [54], the usefulness of IL-6 and CRP as biomarkers of SSVD, particularly in the presence of WML, remains to be fully established [48]. In contrast, CSF TNF-α, TGF-β and vascular endothelial growth factor (VEGF) levels are all elevated in VCI-SSVD patients [55, 56].

CSF *α1-antichymotrypsin*, an acute phase inflammatory protein, was also increased in individuals with VCI-SSVD compared to healthy controls as well as in prodromal VCI-SSVD patients who later progressed to dementia, whereas in AD it was elevated only in manifest dementia [57]. Interestingly, higher levels of serum α1-antichymotrypsin in individuals with VCI, but not AD, compared to controls have also been reported [58].

*YKL-40*, a marker of glial activation, was elevated in CSF of patients with prodromal VCI but not prodromal AD [59].

### Markers of extracellular matrix breakdown

Matrix metalloproteinases (MMPs) are a large family of enzymes active in the extracellular matrix, at the cell surface and intracellularly. Although there are 26 family members, MMP-2, −3, −7, −9, −10 and −12 are mainly active in the brain. Some MMPs, i.e. MMP-2, are constitutively produced and are normally present in the CSF. Others (mainly MMP-3 and MMP-9) are inducible with very low levels in the CSF until an inflammatory response is elicited [60, 61]. Changes in several MMPs have been found in SSVD [33]. Measurement of MMPs and tissue inhibitor of metalloproteinases-1 (TIMP-1) are promising SSVD biomarkers, and have high validity in discriminating VCI-SSVD from cognitive impairment of primarily neurodegenerative etiology [33, 34, 57, 62, 63].

### Markers of subcortical neuronal degeneration and myelin damage

#### Neurofilaments

Neurofilaments (NFs) are major structural proteins of neurons. They consist of three subunits of low (NF-L), medium (NF-M), or high (NF-H) molecular weight with varying degrees of phosphorylation [64]. NFs may be sensitive surrogate markers for neuronal death and axonal loss [65]. Several early studies detected NF peptides in the CSF of several neurological/neurodegenerative disorders [66–69]. NF-L subunit is a protein expressed in large-caliber myelinated axons [70]. Slightly increased CSF NF-L levels occur in healthy older individuals and correlate with increasing age [66]. However, a more significant increase in CSF NF-L levels is also present in individuals with WML [32, 33, 71, 72]. A positive association of CSF-NF-L levels with increasing severity of WML in non-demented subjects has also been reported [73].

In acute cerebral infarction, very high NF-L levels were reported [66]. The CSF levels of NF-L were higher in dementia disorders engaging subcortical brain regions, such as VCI and MD, but also in fronto-temporal dementia (FTD) [74, 75]. In VCI-SSVD the CSF NF-L concentrations were consistently higher than in controls, however with considerable overlap among other dementia disorders [32, 72]. In a recent meta-analysis comparing 106 VCI-SSVD patients with 283 healthy age-matched controls, the patient group had increased CSF NF-L levels. However, the overall elevation was smaller than that for either AD or FTD patients versus controls [76].

Increased WML load and ventricular dilation were related to increased CSF levels of TIMP-1 and NF-L and to decreased sAPPβ (a marker of amyloid pathology), suggesting that these molecules may function as biological markers of white matter damage [77].

Far less is known about CSF levels of the other NF isoforms. One study reported increased CSF levels of phosphorylated NF-H/M in AD compared with VCI and controls [67]. Others have found elevated CSF NF-H levels in AD and VCI in comparison with controls, but no differences between FTD and controls, or between AD, VCI and FTD patients [78].

#### Myelin basic protein

Myelin basic protein (MBP) is a major structural constituent of the myelin sheath [79]. Its function is to maintain the structure of the myelin and together with myelin-associated glycoprotein (MAG) to modulate the caliber of myelinated axons [80]. One of the hallmarks of VCI-SSVD is the rarefaction of white matter, due to nerve fiber degeneration, gliosis, demyelination or a combination of all three [81, 82]. Significantly elevated MBP CSF levels have been reported in stroke with subcortical infarcts affecting the white matter, as opposed to stroke with cortical infarcts [83], indicating its potential as a regional marker of infarction, as well as a marker of WML. Increased CSF levels of MBP and NF-L were found in acute ischemic stroke patients. In mild stroke {NIHSS <5 (National Institute of Health Stroke Scale)}, the concentration of MBP was significantly lower compared to more severe stroke (NIHSS >5), while NF-L was a stronger marker for stroke in general, independent of severity [84]. Increased CSF levels of MBP and NF-L were also described in patients with WML as compared to controls. However, compared to controls, MBP and NF-L were increased in both VCI-SSVD and AD patients, with considerable overlap between patient groups [33].

#### CSF sulfatide

CSF sulfatide is an acidic glycophospholipid of oligodendrocyte-produced myelin sheaths considered as a marker of white matter degradation. It was found to be 200% higher in patients with VCI-SSVD compared to controls and patients with AD [85], while CSF sulfatide levels distinguished between patients with subcortical arteriosclerotic encephalopathy and those with normal pressure hydrocephalus with a sensitivity and specificity of 74 and 94% respectively [86]. CSF sulfatide has also been shown to predict WML progression in nondisabled patients with WML [87].

Furthermore, this marker was lower in mild cognitive impairment and mild AD compared to control subjects [88]. In another study TNF-α levels were significantly correlated with sulfatide levels [89], suggesting that this apoptosis-inducing cytokine may lead to oligodendrocytes death, thus contributing to white matter degeneration, a hallmark of SSVD.

### Markers of cortical neuronal degeneration

#### Tau proteins

Tau protein is the major component of intracellularly located neurofibrillary tangles and, in the neurofibrillary pathology, it is present in a hyperphosphorylated form. Both total and hyperphosphorylated tau have been found to be increased in the CSF of patients with AD [90]. Total tau (T-tau) is viewed as a marker of neuronal and/or axonal degeneration, while hyperphosphorylated tau (P-tau) is a more specific marker of tangle formation in AD [91]. Although their diagnostic accuracy may be reduced to variable degree when attempting differentiating AD from other types of dementia, the above biomarkers, especially when combined with Aβ42, achieve sensitivities and specificities >90% for the discrimination of AD at least from normal ageing. They have now been incorporated in *research* guidelines for diagnosing incipient and manifest AD [92, 93].

In VCI, CSF T-tau levels have been reported to be either normal [94–96], increased [97–101], or intermediate between those found in controls and AD, but much lower as compared to those of AD [102, 103]. Even then, some patients with VCI do present with high or, sometimes, very high T-tau levels [96, 103–106]. When patients with VCI, MD or AD with WML were clinically separated, the results were again conflicting: T-tau in VCI was reported as comparable to controls [96], increased [101] or intermediate but much lower in comparison to those of AD [103], while patients with MD presented with increased T-tau in all studies. However, patients with lacunar infarcts [94], progressive WML [99] or VCI-SSVD (pure and/or combined with AD) [32, 33, 107, 108] had normal T-tau levels. The CSF levels of P-tau have been described as normal in VCI or VCI-SSVD [33, 100, 101, 103, 107, 108], while in MD, levels were increased to the level of AD [103] or intermediate between controls and AD [107].

### Markers of amyloid pathology

#### β-amyloid

Beta-amyloid peptides with 40 (Aβ40) and, especially, with 42 amino acids (Aβ42) are the major components of extracellular AD amyloid plaques. Aβ42 is considered to inversely reflect amyloid pathology, having high sensitivity and specificity (>85%) as compared to cognitively intact old subjects and is now recognized as one of the three clinically useful CSF biomarkers (the other two being T-tau and P-tau) for AD [28, 91]. Reduction of CSF Aβ42 in VCI of any type [106] and VCI-SSVD with or without signs of AD [33, 72, 108, 109], at levels similar to AD or intermediate between controls and AD have been reported. In other studies, the levels of Aβ42 in VCI were described as comparable to those of controls and higher than in AD [95, 98, 100, 101, 103], although overlap exists, with some VCI patients presenting with low levels [103]. Most of the above studies agreed that in MD, Aβ42 levels were reduced in a degree comparable to AD. However, the ratio of Aβ42/40, which is reduced in AD, has been found to be comparable to controls in “pure” VCI [110].

#### Amyloid precursor protein β

Soluble amyloid precursor protein β peptide (sAPPβ) is a product of APP cleavage with potential neurotrophic properties on axons [111]. CSF sAPPβ has been shown to correlate with white matter lesion load in CSF [77] and seems to be unaltered in the CSF in AD patients [112].

### Markers of hypercoagulable state

Several plasma markers of coagulation/fibrinolysis have been associated with VCI-SSVD [54]. The clotting cascade is regulated by balance of activators and inhibitors. Activators may be raised in hypercoagulable state either independently or in parallel with the lowering of inhibitors. The validation of candidate biomarkers is complicated by the existence of heterogeneity among cerebral vessels in different brain regions in response to coagulation dysfunction [113]. In addition, a tenable association with VCI-SSVD has often been difficult to demonstrate with certainty for most proposed markers [114]. Clinical observations have been strengthened, at least in part, by a recent demonstration of a number of downregulated coagulation-related genes in SSVD, after postmortem gene-expression microarray analysis [115].

#### Markers of coagulation cascade

*Fibrinogen* is the endpoint plasma protein of the clotting cascade. Conflicting results have been published regarding its possible significance as a biomarker in VCI-SSVD [116–122].

*Factor VII* (also known as serum prothrombin conversion accelerator) has been found increased [123], whereas *antithrombin III*, a plasma protein that inactivates thrombin, and *D-dimer*, a fibrin degradation product, were found to be reduced in VCI-SSVD [113, 114, 124].

#### Markers derived from the endothelial cells, nearby tissue, or platelets

Many studies report that numerous proteins expressed by the endothelial cells are positively associated with lacunar infarcts or WML and thus increased in VCI-SSVD because of endothelial damage [113, 123, 125–128]. There is a still growing list of these molecules, which includes: *von Willebrand factor* (vWF) [123]; *thrombomodulin* (CD141 or BDCA-3); *the monokine induced by γ-interferon* (MIG) or *chemokine (C-X-C motif) ligand 9* (CXCL-9) [129]; *soluble intercellular adhesion molecule-1* (sICAM-1 also known as CD54); *soluble vascular cell adhesion molecule-1* (sVCAM-1); and *soluble E-selectin* (sE-selectin), the two latter mediating the adhesion of white cells (except neutrophils) to the vascular endothelium [130–133]. However, other neuropathology studies did not confirm a local endothelial activation, and showed that the endothelial layer remains intact in VCI-SSVD [134, 135]. Specifically, the local expression of ICAM-1 and thrombomodulin in the vascular endothelium of small arteries was not confirmative of an association with VCI-SSVD [136]. Thus, the usefulness of these markers for diagnostic purposes remains to be established, as recently reviewed [137].

### Other neurotoxic/metabolic biomarkers

*Homocysteine* (Hcy) is the metabolic product of dietary methionine. Hcy is also synthesized in the liver and kidney. Increased total Hcy plasma levels is associated with risk, clinical deterioration and severity of WML in symptomatic patients with SSVD [138, 139]. Still, the links between hyperhomocysteinemia and SSVD (including a possible endothelial mechanism) are poorly understood [138]. As recently reviewed, when Hcy plasma levels are below 100 μM vascular effects are primarily seen, whereas adverse effects on the nerve cells appear, only when concentrations exceed 100 μM (above clinically-relevant range) [140]. Still we are far from a full identification and characterization of the key molecular pathways linking Hcy to VCI-SSVD. However, preclinical and clinical data support the notion that Hcy is an important mediator of VCI-SSVD.

*B-type natriuretic peptide* (BNP, also referred to as ventricular natriuretic peptide) is a polypeptide secreted by heart ventricles in response to excessive stretching of cardiomyocytes. Similarly, cardiac troponin T is a very sensitive and specific indicator of myocardial damage. A few studies report that serum levels of BNP [141, 142] or cardiac troponin T [143] are elevated in VCI-SSVD. These results need to be substantiated by other investigations to propose these molecules as relevant biomarkers for VCI-SSVD.

### Oxidative stress markers

Clinical studies on markers of oxidative stress in patients with VCI-SSVD and cognitive impairment are scarce. The Framingham study reported lower plasma levels of *myeloperoxidase* in participants with greater WML volumes and silent brain infarcts [47]. *Asymmetric dimethylarginine* (ADMA) is a key chemical involved in normal endothelial function and thus cardiovascular health. Higher plasma ADMA levels were associated with an increased prevalence of silent brain infarcts after adjustment for traditional stroke risk factors, indicating its potential usefulness as a new biomarker of subclinical vascular brain injury [144]. Patients with large-vessel disease had higher oxidative stress (as measured by the serum levels of thiobarbituric acid-reactive substances), but lower antioxidant defense (as measured by serum levels of free thiol) compared to those with SSVD after an acute ischemic stroke [145].

## Results and discussion

### Overlapping and differentiating biomarker findings in VCI-SSVD and AD

Hitherto, there are no yet established biochemical markers for VCI-SSVD, although there are potential candidates. For AD, CSF T-tau, P-tau and Aβ42 have been included in the diagnostic criteria by the National Institute on Aging and the Alzheimer’s Association workgroup for all phases of AD, published in 2011 [92]. By analogy, in VCI-SSVD a major step forward will be to stratify biochemically homogenous patient groups. From the evidence presented in the current work, a significantly elevated albumin ratio reflecting BCB/BBB dysfunction, a hallmark of VCI-SSVD, is a consistent finding in studies with VCI-SSVD and MD [32, 33, 146, 147]. In AD, the albumin ratio is not different from that of controls [33, 72, 147, 148].

Other promising biomarkers are those reflecting damage to the white matter, such as CSF NF-L and MBP. Both markers correlate with SSVD, but they have also been found to be elevated in AD. This overlap possibly reflects concomitant AD and SSVD pathology. However, both CSF NF-L and MBP can be of value in detecting damage to white matter in patients with VCI-SSVD or MD regardless of the white matter etiology. Markers, such as sulfatide, sAPPβ, MMP-2, MMP-3, MMP-9 and TIMP-1, also hold potential for VCI-SSVD biomarkers, though some of them (MMP-2, MMP-3 and MMP-9) have also been described to be altered in AD [33, 57, 62, 63, 77, 85–87, 149, 150]. Other glial and neuronal markers, such as YKL-40, GFAP, S100B and NSE, are also related to AD and are thus not specific [41, 42, 151–153]. Markers of inflammation (i.e. IL-6, TNF-α, TGF-β, VEGF, sICAM-1, sVCAM and sE-selectin) have all been found to be increased in both diseases [56, 154, 155]. Other markers of questionable specificity for SSVD, as compared to AD, are CRP, ADMA and Hcy, since they have also been found to be elevated in AD [57, 155]. Biomarkers closely related to alterations of the vessel wall (vWF, thrombomodulin) and partakers in the coagulation/fibrinolysis system (D-dimer, Factor VII, antithrombin III), rather than inflammation in general, may prove to be more specific for VCI-SSVD and need to be investigated in the context of AD differential diagnosis. It should be noted that the overlap among the above-mentioned biomarkers might be a result of heterogeneous AD populations containing MD patients emphasizing the need for further investigations of “pure” VCI-SSVD and AD cases.

At present, combination of markers reflecting SSVD with markers that seem to be more specific for AD pathology such as Aβ42, T-tau and especially P-tau, appears promising for the exclusion of pure AD or favoring the diagnosis of MD [33].

### Are biomarkers useful for detection of VCI –SSVD in clinical practice?

Thus far, biochemical markers with potential benefit for the diagnosis of VCI-SSVD have limited use in every day clinical practice (see Table 1). Since combination of biomarkers increases accuracy of the AD diagnosis, it seems feasible that a multimodal biomarker approach may be beneficial for the diagnosis of VCI-SSVD as well. In the Ten Point Scale of Binswanger’s disease (which is synonymous with VCI-SSVD), three axes of biomarkers are included: biochemical, imaging and clinical [34, 156]. Increased albumin ratio, reduced MMP-2, elevated NF-L in the absence of a characteristic AD CSF profile are emphasized as the most significant among biochemical markers [34, 156]. Individuals with the highest scores, i.e. presence of the most of the aforementioned biomarkers, are most likely to have VCI-SSVD. Of course, this scoring system requires validation and standardization in large multi-centers longitudinal studies.

**Table 1:** Summary of studies on candidate CSF biomarkers for VaD, VCI and SSVD according to their discrimination power (vs. Controls and/or AD)

Candidate biomarker	Study [Ref]	Date	Change	HC *n*	NC *n*	VaD *n*	VCI *n*	SSVD *n*	AD *n*	Difference vs. Controls	Difference vs. AD	Sn/Sp vs. Controls	Sn/Sp vs. AD
Albumin ratio (CSF/serum)	Wallin et al. [30]	1990	↑	30	-	53	-	-	-	*P* < 0.001	-	-	-
	Bjerke et al [72]	2009	→	52	-	-	-	9	20	NS	NS	-	-
	Bjerke et al. [33]	2011	↑	30	-	-	-	26	30	*P* < 0.005	*P* < 0.01	-	-
	Wallin et al. [32]	2001	↑	18	-	-	-	25	-	*P <* 0.001	-	-	-
NSE	Blennow et al. [41]	1994	↑	33	-	19	-	-	45	*P* < 0.0001	NS	-	-
TNF-α	Tarkowski et al. [55]	1999	↑	25	-	33	-	-	34	*P* < 0.001	-	-	-
VEGF	Tarkowski et al. [56]	2002	↑	27	-	26	-	-	20	*P* = 0.03	NS	-	-
TGF-beta	Tarkowski et al. [56]	2002	↑	27	-	26	-	-	20	*P* < 0.0004	NS	-	-
YKL-40	Olsson et al. [59]	2013	↑	-	65	19	-	-	-	*P* < 0.05	-	-	-
MMP-9	Adair et al. [62]	2004	↑	8	-	15	-	-	30	*P* < 0.003	*P* < 0.0001	-	-
	Bjerke et al. [33]	2011	↑	30	-	-	-	26	30	*P* < 0.05	*P* < 0.05	-	-
MMP-10	Bjerke et al. [33]	2011	↑	30	-	-	-	26	30	*P* < 0.005	NS	-	-
MMP-2	Bjerke et al. [33]	2011	→	30	-	-	-	26	30	NS	NS	-	-
MMP-3	Bjerke et al. [33]	2011	→	30	-	-	-	26	30	NS	NS	-	-
TIMP-1	Ohrfelt et al. [57]	2011	↑	52	-	-	-	7	15	*P* = 0.01	-	-	-
	Ohrfelt et al. [57]	2011	↑	52	-	-	-	8	24	*P* = 0.03	-	-	-
	Bjerke et al. [33]	2011	↑	30	-	-	-	26	30	*P* < 0.05	*P* < 0.005	-	-
TIMP-2	Bjerke et al. [33]	2011	→	30	-	-	-	26	30	NS	NS	-	-
NF-L	Wallin et al. [32]	2001	↑	18	-	-	-	25	-	*P* < 0.001	-	Sn 68% Sp 85%	-
	Bjerke et al. [72]	2009	↑	52	-	-	-	9	20	*P* < 0.001	NS	-	-
	Bjerke et al. [33]	2011	↑	30	-	-	-	26	30	*P* < 0.0001	*P* < 0.05	-	-
MBP	Bjerke et al. [33]	2011	↑	30	-	-	-	26	30	*P* < 0.0001	*P* < 0.005	-	-
Sulfatide	Fredman et al. [85]	1992	↑	19	-	20	-	-	43	*P* < 0.0001	*P* < 0.0001	-	-
Total tau (τ_T_)	Wallin et al. [32]	2001	→	18	-	-	-	25	-	NS	-	Sn 85% Sp 36%	-
	Paraskevas et al. [103]	2009	↑	68	_-	23	-_	-_	92	*P* < 0.05	*P* < 0.05	-_	Sn 80% Sp 86%
	Bjerke et al. [72]	2009	→	52	-	-	-	9	20	NS	*P* < 0.005	-	-
	Bjerke et al. [33]	2011	↑	30	-	-	-	26	30	*P* < 0.005	NS	-	-
Phospho-tau (τ_p-181_)	Paraskevas et al. [103]	2009	→	68	-_	23	_-	-_	92	NS	*P* < 0.01	-_	Sn 84% Sp79%
	Bjerke et al. [72]	2009	→	52	-	-	-	9	20	NS	*P* < 0.005	-	-
	Bjerke et al. [33]	2011	→	30	-	-	-	26	30	NS	*P* < 0.05	-	-
Aβ_42_	Paraskevas et al. [103]	2009	→	68	_-	23	-_	_-	92	NS	NS	-_	Sn 73% Sp 70%
	Bjerke et al. [72]	2009	↓	52	-	-	-	9	20	*P* < 0.05	*P* < 0.005	-	-
	Bjerke et al. [33]	2011	↓	30	-	-	-	26	30	*P* < 0.001	NS	-	-
Aβ_42_, τ_T,_ τ_p-181_ (combination)	Paraskevas et al. [103]	2009	↑	68	-_	23	-_	_-	92	NS	*P* < 0.01	_-	Sn 87% Sp 89%
sAPP_β_	Bjerke et al. [77]	2014								Correlation with WML	-	-	-

*VaD* Vascular Dementia, *VCI* Vascular Cognitive Impairment, *SSVD* Subcortical Small Vessel Disease, *AD* Alzheimer’s disease, *HC* Healthy Controls, *NC* Neurological Controls, *Sn/Sp* Sensitivity/Specificity, *n* number of controls/patients, *↓* reduced levels, ↑ increased levels, → no difference vs. controls, − not included, *NS* Not Significant

## Conclusion and future directions

VCI-SSVD is a common neurocognitive disorder with similar clinical manifestations similar to other disorders, such as AD. Underestimation of the impact of SSVD on cognition and insufficient knowledge of SSVD in AD may explain why VCI-SSVD is under-recognized in clinical practice and not often studied in research contexts. Biochemical markers may be of help for the (differential) diagnosis of VCI-SSVD. They can also be used for identifying patients with preclinical SSVD from apparently healthy controls. Furthermore, they have a potential to define a spectrum disorder with pure subcortical vascular disease (i.e. Binswanger’s disease) at one end of the spectrum and pure AD at the other. In between will be the large group of MD patients that will remain a major diagnostic challenge.

This review of all biological markers studied in patients with VCI-SSVD identified several fluid biomarkers with evidence for use in diagnostic settings, while others are too early to be considered as potential SSVD biomarkers. There is little value of blood tests at this time since none of the above biomarkers have been adequately studied, whereas CSF helps to separate vascular and neurodegenerative causes based on the presence of BCB/BBB disruption and extracellular matrix breakdown. Obviously, many of the CSF reports cited in this review are based on small series of cases. Replication studies should be done to ensure the reproducibility of the results and calculation of positive and negative predictive values should be provided. At present, it is unlikely that single markers may be sufficient for diagnostic and differentiating purposes. Instead, a combination of biochemical and imaging markers as well as psychometrics will be necessary to improve the diagnostic accuracy.

A third potential use of fluid biomarkers is their application as surrogates for disease progression e.g. in clinical pharmacological trials. For that purpose, such biomarkers could be used if different levels of the biomarkers can reflect patients’ clinical severity or extent of WML. If a biomarker fulfills this definition, it could be very valuable in clinical trials obviating the need for repeated expensive MRI examinations.

A fourth potential use of fluid biomarkers is to clarify the pathogenesis of the disorder, for example to verify an inflammatory underlying process, perhaps in a subgroup of patients, thus suggesting a (novel) target for therapy. Clearly, much further work needs to be done along these lines.

The search for an optimal panel of biomarkers with high sensitivity and specificity through a collaborative international network of biobanks, multi-center collections based on large patient cohorts, combined with population genetics, clinical trials and harmonized protocols and procedures will provide the crucial tools needed to enhance the likelihood of success in identifying valid biomarkers in VCI-SSVD [157]. In addition, research will benefit from innovative statistical approaches that allow handling large datasets, e.g. strategies used in the field of artificial intelligence. With combined efforts, the development of biomarkers in the VCI-SSVD field may not only foster therapeutic progress in VCI-SSVD but also in AD.

## Abbreviations

AD: Alzheimer’s disease
ADMA: Asymmetric dimethylarginine
Aβ40: Beta-amyloid peptide with 40 amino acids
Aβ42: Beta-amyloid peptide with 42 amino acids
BBB: Blood–brain barrier
BCB: Blood-cerebrospinal fluid barrier
BNP: B-type natriuretic peptide
CRP: C reactive protein
CSF: Cerebrospinal fluid
CT: Computed tomography
CXCL-9: Chemokine (C-X-C motif) ligand 9
FTD: Fronto-temporal dementia
GFAP: Glial fibrillary acidic protein
Hcy: Homocysteine
IL-6: Interleukin-6
ILs: Interleukins
MAG: Myelin-associated glycoprotein
MBP: Myelin basic protein
MD: Mixed type dementia
MIG: Monokine induced by γ-interferon
MMPs: Matrix metalloproteinases
MRI: Magnetic resonance imaging
NF-H: Neurofilament heavy subunit
NF-L: Neurolfilament light subunit
NF-M: Neurofilament medium subunit
NFs: Neurofilaments
NIHSS: National institute of health stroke scale
NSE: Neuron specific enolase
P-tau: Hyperphosphorylated tau
QA: Cerebrospinal fluid/serum albumin quotient
sAPPβ: soluble amyloid precursor protein β peptide
sE-selectin: soluble E-selectin
sICAM-1: soluble intercellular adhesion molecule-1
SSVD: Subcortical small vessel disease
sVCAM-1: soluble vascular cell adhesion molecule-1
TIMP-1: Tissue inhibitor of metalloproteinases-1
T-tau: Total tau
VCI: Vascular cognitive impairment
VCI-SSVD: Vascular cognitive impairment of the subcortical small vessel disease type
WML: White matter lesions
